# Novel Benzoxazine-Based Aglycones Block Glucose Uptake *In Vivo* by Inhibiting Glycosidases

**DOI:** 10.1371/journal.pone.0102759

**Published:** 2014-07-21

**Authors:** Hanumantharayappa Bharathkumar, Mahalingam S. Sundaram, Swamy Jagadish, Shardul Paricharak, Mahadevappa Hemshekhar, Daniel Mason, Kempaiah Kemparaju, Kesturu S. Girish, Basappa, Andreas Bender, Kanchugarakoppal S. Rangappa

**Affiliations:** 1 Laboratory of Chemical Biology, Department of Chemistry, Bangalore University, Bangalore, India; 2 Department of Studies in Biochemistry, University of Mysore, Mysore, India; 3 Department of Chemistry, University of Mysore, Manasagangotri, Mysore, India; 4 Unilever Centre for Molecular Science Informatics, Department of Chemistry, University of Cambridge, Cambridge, United Kingdom; 5 Division of Medicinal Chemistry, Leiden Academic Centre for Drug Research, Leiden University, Leiden, The Netherlands; University of East Anglia, United Kingdom

## Abstract

Glycoside hydrolases catalyze the selective hydrolysis of glycosidic bonds in oligosaccharides, polysaccharides, and their conjugates. β-glucosidases occur in all domains of living organisms and constitute a major group among glycoside hydrolases. On the other hand, the benzoxazinoids occur in living systems and act as stable β-glucosides, such as 2-(2,4-dihydroxy-7-methoxy-2H-1,4-benzoxazin-3(4H)-one)-β-D-gluco-pyranose, which hydrolyse to an aglycone DIMBOA. Here, we synthesized the library of novel 1,3-benzoxazine scaffold based aglycones by using 2-aminobenzyl alcohols and aldehydes from one-pot reaction in a chloroacetic acid catalytic system via aerobic oxidative synthesis. Among the synthesized benzoxazines, 4-(7-chloro-2,4-dihydro-1H-benzo[d][1,3]oxazin-2-yl)phenol (compound 7) exhibit significant inhibition towards glucosidase compared to acarbose, with a IC_50_ value of 11.5 µM. Based upon results generated by *in silico* target prediction algorithms (Naïve Bayesian classifier), these aglycones potentially target the additional sodium/glucose cotransporter 1 (where a log likelihood score of 2.70 was observed). Furthermore, the *in vitro* glucosidase activity was correlated with the *in silico* docking results, with a high docking score for the aglycones towards the substrate binding site of glycosidase. Evidently, the *in vitro* and *in vivo* experiments clearly suggest an anti-hyperglycemic effect via glucose uptake inhibition by 4-(7-chloro-2,4-dihydro-1H-benzo[d][1,3]oxazin-2-yl)phenol in the starved rat model. These synthetic aglycones could constitute a novel pharmacological approach for the treatment, or re-enforcement of existing treatments, of type 2 diabetes and associated secondary complications.

## Introduction

Diabetes mellitus, particularly its subtype 2 (T2-DM), is considered to be a major and increasing threat to human health and accounts for an estimated more than 300 million cases of diabetes worldwide [1], [2]. Diabetes is associated with a significant number of co morbidities, such as cardiovascular disorders, stroke, diabetic retinopathy and kidney dysfunction, and long-term complications may even lead to limb amputations, among other consequences [2].

In addition to injectable insulin and analogs thereof, four distinct classes of oral hypoglycemic agents are currently used for the treatment of T2-DM. In addition to metformin as an oral drug used for the early control of T2-DM, there are a number of second- and third-line pharmacological agents available, such as sulfonylureas, thiazolidinediones, incretin-based remedies, and α-glucosidase inhibitors. Given the increased perception that handling the early stages of diabetes is of crucial importance, several recent studies and approaches are focusing on agents that can delay or inhibit glucose absorption. Delaying glucose absorption, such as by blocking glycoside hydrolases (particularly α-glucosidases), allows extended time for β-cells to increase insulin secretion, and thereby reduce circulatory glucose levels [3], [4].

α-glucosidases are membrane-bound enzymes that catalyze the selective hydrolysis of glycosidic bonds in oligosaccharides, polysaccharides, and their conjugates to release glucose and the respective monosaccharides. Although they occur throughout living organisms, most of them are located in the brush border of the small intestine to facilitate glucose uptake [4], [5]. Thus, the use of α-glucosidase inhibitors (AGIs) for the treatment of T2-DM delays the release of glucose and halts glucose absorption, thereby lowering the postprandial blood glucose level and improving pre-diabetic conditions. The currently most prominent AGIs are acarbose (Glucobay), a natural compound from an Actinoplanes strain, and the N-hydroxyethyl analogue of 1-deoxynojirimycin, miglitol [4], [6]. However, these compounds have been reported to cause severe gastrointestinal complications.

**Figure 1:**
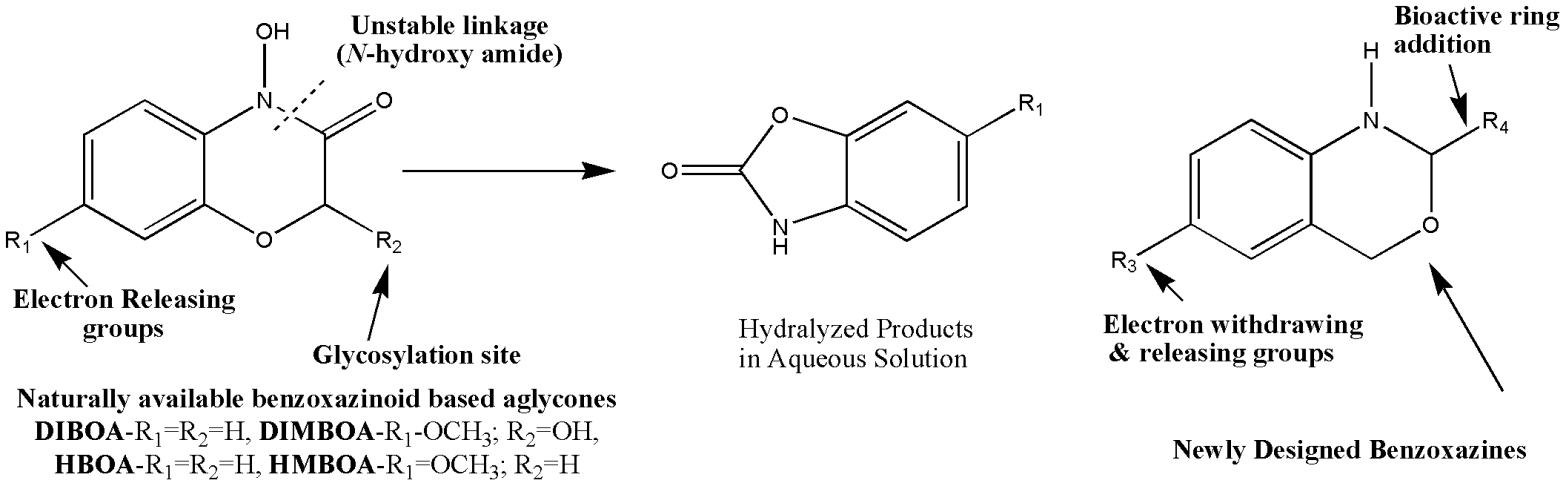
Scheme for the proposed strategy for the synthesis of 1,3-benzoxazine derivatives.

Numerous efforts have hence been made to further develop AGIs in order to improve treatment of pre-diabetic states [7], [8]. Glycosides, the natural substrates for the glycoside hydrolases which are composed of an aglycone and a glycan moiety, have recently attracted particular attention in this field. Stable β-glucosides such as 2-(2,4-dihydroxy-7-methoxy-2H-1,4-benzoxazin-3(4H)-one)-β-D-gluco-pyranoside and desmethoxy derivatives including 6,7-dimethoxy-benzoxazolin-2(3H)-one, 4-hydroxy-2H-1,4-benzoxazin-3(4H)-one, and 4-acetylbenzoxazolin-2(3H)-one, are found in living plants [9]. These glucosides are biologically inactive, but enzymatically converted to active aglycones such as DIMBOA (2,4-dihydroxy-7-methoxy-2H-1,4-benzoxazin-3(4H)-one and the desmethoxy derivative DIBOA, by β-glucosidases [10]. These products have diverse effects, that include anti-auxin, anti-inflammatory, and powerful antibiotic activities. These aglycones are further degraded spontaneously to the corresponding benzoxazolinones, MBOA (6-methoxy-benzoxazolin-2(3H)-one) and the desmethoxy derivative BOA. Degradation is faster if the benzene ring and amide linkage bears electron donating or hydroxyl groups [11], and these chemical variations are what we explored in more detail in the current work. In addition to this, our aim was to remove the relatively labile N-hydroxy amide moiety *via* replacement by a suitable ring system, masking the glycosylation site of glycoside (**Fig. 1**). These efforts have the goal of increasing efficacy of the compounds *in vivo*, in order to lower blood sugar levels by inhibiting glucosidase.

## Materials and Methods

### Chemicals/ Reagents

Rat intestinal acetone powder was purchased from Sigma Aldrich, St. Louis (USA), Acarbose was purchased from Glucobay Bayer AG (Germany). Porcine pancreatic α-Amylase and Yeast α-Glucosidase were purchased from SRL, Mumbai (India). Glucose oxidase (GOD POD) kit was purchased from Piramal HealthCare Ltd, Mumbai (India). All other chemicals used were of analytical grade and purchased from Sigma Aldrich, St. Louis (USA) and SRL, Mumbai (India). All IR spectra were obtained in KBr disc on a Shimadzu FT-IR 157 Spectrometer. ^1^H and ^13^C NMR spectra were recorded on a Bruker WH-200 (400 MZ) spectrometer in CDCl_3_ or DMSO-d_6_ as solvent, using TMS as an internal standard and chemical shifts are expressed as ppm. Mass spectra were determined on a Shimadzu LC-MS. The elemental analyses were carried out using an Elemental Vario Cube CHNS rapid Analyzer. The progress of the reaction was monitored by TLC pre-coated silica gel G plates.

### Experimental animals

Adult Wistar rats weighing 150–180 g were collected from the University Central Animal Facility and housed under a controlled environment. All animal experiments were approved by the Institutional Animal Ethical Committee (Order No : MGZ/2620/2011-12 Dated 31-01-2012; UOM/IAEC/18/2011), Department of Studies in Zoology, University of Mysore, Mysore and were in accordance with the guidelines of the Committee for the Purpose of Control and Supervision of Experiments on Animals (CPCSEA).

### Synthesis and characterization of 1,3-benzoxazine derivatives

**Figure 2:**
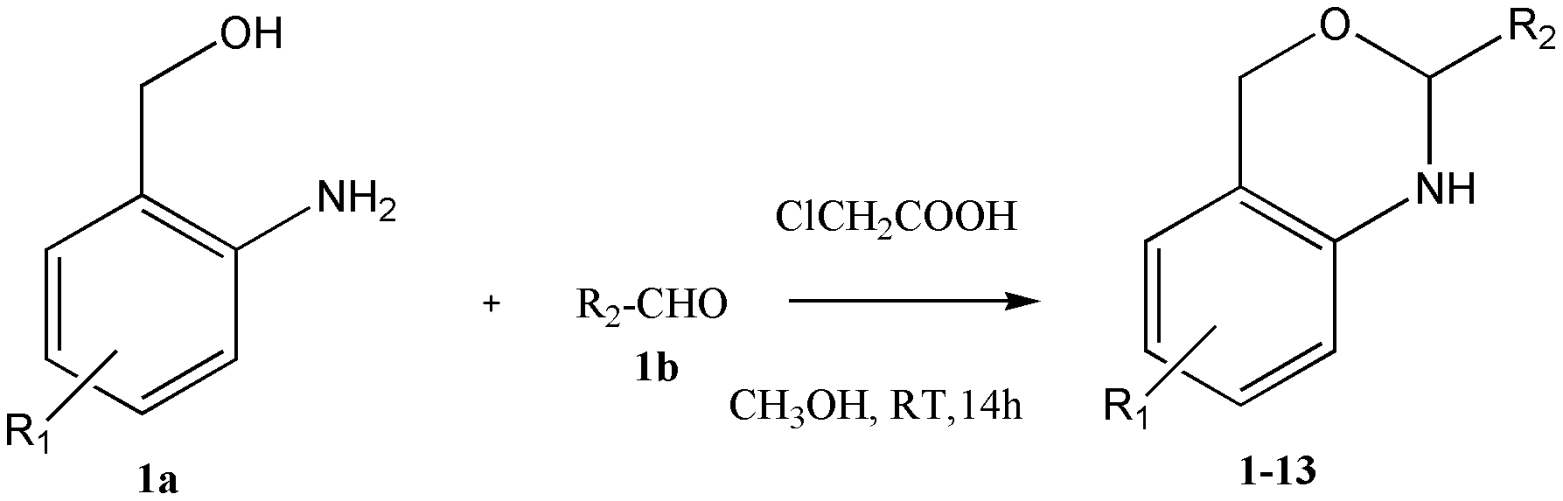
General procedure for the synthesis of novel benzoxazines.

This work is based on the previous synthesis of an oxazine derivative which was able to mimic the pyranoside structure of glycans functionally [12]. In continuation of the glycobiological aspects, the one-pot syntheses of a novel 1,3-benzoxazine scaffold was carried out using 2-aminobenzyl alcohols and different aldehydes in chloroacetic acid *via* aerobic oxidative synthesis (Fig. 2).

#### General Procedure for the Synthesis of benzoxazines

To a 50 mL round bottom flask, amino benzyl alcohol (1.0 equiv), aldehyde (1.0 equiv), chloroacetic acid (1.0 equiv) and methanol (10 mL) were added. The reaction mixture was then stirred at room temperature for 16 h. Water was added to the reaction mixture, and extracted with ethyl acetate. The organic layer was dried by using anhydrous sodium sulphate, and then concentrated in vacuum to afford compound benzoxazine as a crystalline solid. The compounds were then purified by column chromatography and characterized *via* IR, ^1^H NMR, ^13^C NMR, mass spectrometry and Elemental Analysis.

#### Synthesis of 4-(2,4-dihydro-1H-benzo[d][1,3]oxazin-2-yl)phenol 1

The product **1** was obtained from 2-amino benzyl alcohol (1.23 g,10 mmol), 4-hydroxy benzaldehyde (1.22 g, 10 mmol) and chloroacetic acid 0.94 g (10 mmol). This compound was obtained as brownish solid in 83% yield. IR νmax (KBR): 3277,2924, 1514, 1220 cm^−1^, **^1^H NMR** (400 MHz, CDCl_3_ δ in ppm) δ 4.13(1H, s, NH) 4.80-4.90 (2H, s, CH_2_) 5.02 (1H, s, O-H) 5.28 (1H, s,-CH) 6.70-6.72(1H, d, Aromatic -CH) 6.9–7.2 (5H, m, Ar-CH) 8.06 (1H, s, Ar-CH); **^13^C NMR** 70.05, 97.95, 114.64, 120.47, 126.08, 126.13, 129.13, 129.79, 130.22, 138.07, 147.90, 155.51. **MASS**; m/z found for C_14_H_13_NO_2_ 228.2 ([M+1]^+^).

#### Synthesis of 2-(1H-indol-3-yl)-2,4-dihydro-1H-benzo[d][1,3]oxazine 2

The product **2** was obtained from 2-amino benzyl alcohol (1.23 g, 10 mmol), indole-3-carbaldehyde (1.45g, 10 mmol) and chloroacetic acid 0.94g (10 mmol). This compound was obtained as black color solid in 82% yield. IR νmax (KBR): 3252, 2898, 1493, 1244 cm^−1^, **^1^H NMR** (400 MHz, CDCl_3_ δ in ppm) 4.25 (1H,s,NH), 4.70 (2H,s,- CH_2_-) 5.12 (1H,s,-CH-), 7.07-7.50(5H,m, Ar-H), 8.01–8.64(4H,m, Ar-H), 11.80 (1H,s,Indole-NH); Anal. Calcd.for C_16_H_14_N_2_O: C, 76.78; H, 5.64; N, 11.19; found C, 76.54; H, 5.51; N, 11.53 %. **MASS**; m/z found for C_16_H_14_ClN_2_O 251.3 ([M+1]^+^).

#### Synthesis of 2-(2-methyl-1H-indol-3-yl)-2,4-dihydro-1H-benzo[d][1,3]oxazine 3

The product **3** was obtained from 2-amino benzyl alcohol (1.23 g, 10 mmol), 2-methyl indole-3-carbaldehyde (1.59 g, 10 mmol) and chloroacetic acid 0.94g (10 mmol). This compound was obtained as black colour solid in 87% yield. IR νmax (KBR): 3212, 2788, 1462, 1238 cm^−1^, **^1^H NMR** (400 MHz, CDCl_3_ δ in ppm) 2.11(3H,s, -CH_3_-), 4.13 (1H,s,-N-H-), 4.83 (2H,s,-CH_2_-) 5.19 (1H,s-CH-); 7.26(3H,m,-Ar-CH-) 8.06–8.22(5H,m,Ar-CH), 10.12(1H,s,N-H INDOLE) Anal. Calcd.for C_17_H_16_N_2_O; C, 77.25; H, 6.10; N, 10.60; found C, 77.14; H, 6.11; N, 10.43%; **MASS**; m/z found for C_17_H_16_N_2_O 264.4 ([M+1]^+^).

#### Synthesis of 2-(1-(4-(2-cyanophenyl)benzyl-1H-indol-3-yl),2,4-dihydro-1H-benzo(d)(1,3) oxazine 4

The product **4** was obtained from 2-amino benzyl alcohol (1.23 g, 10 mmol), 2-(1-(4-(2-cyanophenyl )1-benzyl-1H-indole-3-carbaldehyde (3.36 g, 10 mmol) and chloroacetic acid 0.94 g (10 mmol). This compound was obtained as brown colour solid in 86% yield. IR νmax (KBR): 3182, 2918, 1473, 1294 cm^−1^, **^1^H NMR** (400 MHz, CDCl_3_ δ in ppm) 4.09 (1H,s, -NH), 4.7 (2H,s,-CH_2_), 5.4 (3H,s,-CH), 5.4 (3H,s,-CH_2_) 7.26–8.35 (17H,m,-Ar-CH-) Anal. Calcd.for C_30_H_23_N_3_O; C, 81.61; H, 5.25; N, 9.52; found C, 81.14; H, 5.11; N, 9.43%. **MASS**; m/z found for C_30_H_23_N_3_O 442.4 ([M+1]^+^).

#### Synthesis of 3-(2,4-dihydro-1H-benzo[d][1,3]oxazin-2-yl)-4H-chromen-4-one 5

The product **5** was obtained from 2-amino benzyl alcohol (1.23 g, 10 mmol), 4-oxo-4H-chromene-3-carbaldehyde (3.21 g, 10 mmol) and chloroacetic acid 0.94 g (10 mmol). This compound was obtained as brownish solid in 89% yield. IR νmax (KBR): 3302, 2918, 1463, 1215 cm^−1^, **^1^H NMR** (400 MHz, CDCl_3_ δ in ppm) 4.11 (1H,s,NH), 4.80 (1H,s,-CH-) 5.32 (2H,s,-CH_2_-), 7.07–8.32(7H,m, Ar-H) 8.55(H,s, Ar-O-CH); **^13^C NMR** 70.1, 85.5, 110.6, 115.2,116.1, 119.6,122.0,127.6, 127,8, 129.2, 132.6, 133.6, 142.2, 149.1,155.6, 179.2; Anal. Calcd. for C_17_H_13_NO_3_: C, 73.11; H, 4.69; N, 5.02; found C, 73.54; H, 4.51; N, 5.23 %; **MASS**; m/z found for C_17_H_13_NO_3_ 278.3 ([M-1]^+^).

#### Synthesis of 2-(2-butyl-4-chloro-1H-imidazol-5-yl)-2,4-dihydro-1H-benzo[d][1,3]oxazine 6

The product **6** was obtained from 2-amino benzyl alcohol (1.23 g, 10 mmol), 2-butyl-4-chloro-1H-imidazole-5-carbaldehyde (1.86 g, 10 mmol) and chloroacetic acid 0.94 g (10 mmol). This compound was obtained as black colour solid in 86% yield. IR νmax (KBR): 3225, 2892, 1433, 1214 cm^−1^ **^1^H NMR** (400 MHz, CDCl_3_ δ in ppm) 0.90 (3H,t,CH_3_), 1.30 (2H,m,- CH_2_-) 1.60 (2H,m,-CH_2_-), 2.60 (2H,t,CH_2_), 4.30 (1H,s,- NH-) 4.60 (2H,s,-CH_2_-), 5.60 (1H,s,- CH-) 6.60–7.60 (4H,m, Ar-H), 8.2 (1H,s, imidazole N-H) Anal. Calcd.for C_15_H_18_ClN_3_O: C, 61.75; H, 6.22; N, 14.40; found C, 61.54; H, 6.51; N, 14.53 %. **MASS**; m/z found for C_15_H_18_ClN_3_O 292.2 ([M+1]^+^).

#### Synthesis of 4-(7-chloro-2,4-dihydro-1H-benzo[d][1,3] oxazin-2-yl)phenol 7

The product **7** was obtained from 2-amino-4-chloro benzyl alcohol (1.57 g, 10 mmol), 4-hydroxy benzaldehyde (1.22 g, 10 mmol) and chloroacetic acid 0.94 g (10 mmol). This compound was obtained as brownish solid in 87% yield. IR νmax (KBR): 3258, 2897, 1487, 1269 cm^−1^ **^1^H NMR** (400 MHz, CDCl_3_ δ in ppm) δ 4.13(1H,s,NH) 4.80-4.90 (2H,s,CH_2_) 5.02 (1H,s,O-H) 5.28 (1H,s,-CH) 6.70-6.72(2H,d, Aromatic -CH) 6.9–7.2 (4H,m,Ar-CH) 8.06 (1H,s,Ar-CH). **^13^C NMR** 68.97, 94.73, 110.80, 115.66, 119.08, 129.97, 130.40, 133.39, 134.24, 139.12, 149.38, 159.12. Anal. Calcd.for C_14_H_12_ClNO_2_; C, 64.25; H, 4.62; N, 5.35; found C, 64.14; H, 4.21; N, 5.53%. **MASS**; m/z found for C_14_H_12_ClNO_2_ 262.2 ([M+1]^+^).

#### Synthesis of 7-chloro-2-(1H-indol-3-yl)-2,4-dihydro-1H-benzo[d][1,3]oxazine 8

The product **8** was obtained from 2-amino-5-chloro benzyl alcohol (1.57 g,10 mmol), indole-3-carbaldehyde (1.45 g, 10 mmol) and chloroacetic acid 0.94 g (10 mmol). This compound was obtained as brownish solid in 85% yield. IR νmax (KBR): 3213, 2978, 1448, 1236 cm^−1^, **^1^H NMR** (400 MHz, CDCl_3_ δ in ppm) 4.25 (1H,s,NH), 4.70 (2H,s,- CH_2_-) 5.12 (1H,s,-CH-), 7.07-7.50(5H,m, Ar-H), 8.01–8.64(3H,m, Ar-H), 11.80 (1H,s,Indole-NH); Anal. Calcd. for C_16_H_13_ClN_2_O: C, 67.49; H, 4.60; N, 9.84; found C, 66.54; H, 4.51; N, 9.53%; **MASS**; m/z found for C_16_H_13_ClN_2_O 285.8 ([M+1]^+^).

#### Synthesis of 6-chloro-2-(2-phenyl-1H-indol-3-yl)-2,4-dihydro-1H-benzo[d][1,3]oxazine 9

The product **9** was obtained from 2-amino-5-chloro benzyl alcohol (1.57 g, 10 mmol), 2-phenyl indole-3-carbaldehyde (2.21 g, 10 mmol) and chloroacetic acid 0.94 g (10 mmol). This compound was obtained as colourless solid in 89% yield. IR νmax (KBR): 3232, 2843, 1452, 1243 cm^−1^, **^1^H NMR** (400 MHz, CDCl_3_ δ in ppm) 2.31(2H,s, -CH_2_-), 2.39 (3H,s,CH_3_), 5.11 (1H,s,-C-H), 4.2 (1H,s,-N-H-) 6.26–8.41 (12H,m,Ar-CH-); 10.00 (1H,s,N-H) Anal. Calcd.for C_22_H_17_N_2_OCl; C, 73.23; H, 4.72; N, 7.76; found C, 73.34; H, 4.31; N, 7.43%; **MASS**; m/z found for C_22_H_17_ClN_2_O 361.5 ([M+1]^+^).

#### Synthesis of 3-(6-chloro-2,4-dihydro-1H-benzo[d][1,3]oxazin-2-yl)-4H-chromen-4-one 10

The product **10** was obtained from 2-amino -5-chloro benzyl alcohol (1.57 g,10 mmol), 4-oxo-4H-chromene-3-carbaldehyde (1.74 g, 10 mmol) and chloroacetic acid 0.94 g (10 mmol). This compound was obtained as brownish solid in 83% yield. IR νmax (KBR): 3219, 2797, 1488, 1285 cm^−1^ **^1^H NMR** (400 MHz, CDCl_3_ δ in ppm) 4.11 (1H,s,NH), 4.80 (1H,s,-CH-) 5.32 (2H,s,-CH_2_-), 7.77–8.55(7H,m, Ar-H); Anal. Calcd.for C_17_H_12_ClNO_3_: C, 65.08; H, 3.86; N, 4.46; found C, 66.54; H, 3.51; N, 4.23%; **MASS**; m/z found for C_17_H_12_ClNO_3_ 314.0 ([M+1]^+^).

#### Synthesis of 6-methyl-2-(2-methyl-1H-indol-3-yl)-2,4-dihydro-1H-benzo[d][1,3]oxazine 11

The product **11** was obtained from 2-amino-5-methyl benzyl alcohol (1.37 g,10 mmol), 2-methyl indole-3-carbaldehyde (1.59 g, 10 mmol) and chloroacetic acid 0.94 g (10 mmol). This compound was obtained as light yellow colour solid in 80% yield. IR νmax (KBR): 3219, 2818, 1513, 1284 cm^−1^, **^1^H NMR** (400 MHz, CDCl_3_ δ in ppm) 2.40(3H,s, -CH_3_-), 2.5 (3H,s,-CH_3_), 4.6 (2H,s,-CH_2_), 5.25 (1H,s,-CH) 6.67–7.32 (7H,m,-Ar-CH-) 10.15(1H,s,N-H), Anal. Calcd.for C_18_H_18_N_2_O; C, 77.67; H, 6.52; N, 10.06; O, 5.75; found C, 77.14; H, 6.11; N, 10.43%. **MASS**; m/z found for C_18_H_18_N_2_O 278.2, 280.2 ([M+1]^+^).

#### Synthesis of 6-methyl-2-(2-phenyl-1H-indol-3-yl)-2,4-dihydro-1H-benzo[d][1,3]oxazine 12

The product **12** was obtained from 2-amino-5-methyl benzyl alcohol (1.37 g, 10 mmol), 2-phenyl indole-3-carbaldehyde (2.21 g, 10 mmol) and chlor acetic acid 0.94 g (10 mmol). This compound was obtained as colourless solid in 86% yield. IR νmax (KBR): 3210, 2817, 1433, 1224 cm^−1^, **^1^H NMR** (400 MHz, CDCl_3_ δ in ppm) 2.31(2H, s, -CH_2_-), 2.39 (3H, s, CH_3_), 5.11 (1H, s, -C-H), 4.2 (1H, s, -N-H-) 6.26–8.41 (12H, m, Ar-CH-); 10.00 (1H, s, N-H) Anal. Calcd. for C_23_H_20_N_2_O; C, 81.15; H, 5.92; N, 8.23; found C, 81.34; H, 5.71; N, 8.43%; **MASS**; m/z found for C_23_H_20_N_2_O 341.2 ( [M+1]^+^).

#### Synthesis of 3-(6-methyl-2,4-dihydro-1H-benzo[d][1,3]oxazin-2-yl)-4H-chromen-4-one 13

The product **13** was obtained from 2-amino-5-methyl benzyl alcohol (1.37 g, 10 mmol), 4-oxo-4H-chromene-3-carbaldehyde (1.74 g, 10 mmol) and chloro acetic acid 0.94 g (10 mmol). This compound was obtained as yellow color solid in 88% yield. IR νmax (KBR): 3241, 2828, 1413, 1224 cm^−1^, **^1^H NMR** (400 MHz, CDCl_3_ δ in ppm) 2.20(3H,s, -CH_3_-), 4.11-4.13 (1H,s,-N-H), 4.11-4.13 (1H, s, -C-H), 4.79 (1H, s, -CH_2_-) 5.26 (1H, s, -CH_2_-); 6.6–8.3 (7H, m, -Ar-CH-) 8.5(1H, s, O-CH), Anal. Calcd. for C_18_H_15_NO_3_; C, 73.71; H, 5.15; N, 4.78; found C, 73.14; H, 5.11; N, 4.43%; **Mass**; m/z found for C_18_H_15_NO_3_ 294.1 ([M+1]^+^).

### Inhibitory activity against α-Amylase

The α-amylase inhibition assay was performed according to a previous report [13]. Porcine pancreatic α-amylase (3 units/mL) was dissolved in 0.1 M phosphate buffered saline, pH 6.9. The various concentrations of 1,3-benzoxazine derivatives (0–100 µM) were pre-incubated with enzyme independently for 10 min at 37°C. The reaction was initiated by adding substrate solution (0.1% starch) to the incubation medium. After 10 min incubation, the reaction was stopped by adding 250 µL dinitrosalicylic (DNS) reagent (1% 3, 5-dinitrosalicylic acid, 0.2% phenol, 0.05% Na_2_SO_3_ and 1% NaOH in aqueous solution) to the reaction mixture. The reaction was terminated by keeping the reaction mixture in boiling water bath for 10 min. Thereafter, 250 µL of 40% potassium sodium tartarate solution was added to the mixtures to stabilize the colour. After cooling to room temperature in a cold water bath, the absorbance was recorded at 540 nm using a Varioskan multimode plate reader (Thermo Scientifics, USA). Acrabose was used as positive control. The percentage of inhibition was calculated using Abs Contol – Abs Sample ×100/Abs Control. Where Abs Control was the absorbance without sample, Abs sample was the absorbance of enzyme with compound. Concentration-response assays were used to determine the potency (IC_50_) of 1,3-benzoxazine derivatives based on the logistic analysis of the concentration-response curve using Microsoft Excel.

### Inhibitory activity against α-Glucosidase

The α-glucosidase inhibitory activities of synthesized compounds were evaluated using the method developed by Tsujii et al. [14]. Briefly, α-glucosidase was dissolved in phosphate buffer (50 mM, pH 6.9) and pre-treated with various concentrations of 1,3-benzoxazine derivatives (0–100 µM) independently for 10 min at 37°C. The reaction was initiated by the addition of 50 µL of 5 mM *p*-nitrophenyl- α -D-glucopyranoside solution in phosphate buffer (50 mM, pH 6.9). The enzyme reaction was carried out at 37°C for 30 min. The reaction was terminated by the addition of Na_2_CO_3_ (1 M). The enzymatic activity of α-glucosidase was quantified by measuring the absorption at 405 nm using a Varioskan multimode plate reader (Thermo Scientifics, USA). The inhibitory effect of compounds was defined as inhibitory activity (%)  =  (Abs Control – Abs Compound treated)/Abs Control ×100. Concentration-response assays were used to determine the potency (IC_50_) of 1,3-benzoxazine derivatives based on the logistic analysis of the concentration-response curve using Microsoft Excel.

### Intestinal α-glucosidase inhibitory assay

Intestinal α-glucosidase activity was determined by measuring the amount of glucose hydrolyzed from maltose or sucrose [15]. Briefly, rat intestinal acetone powder was homogenized in 0.9% saline and the suspension was centrifuged at 10,000 g for 30 min at 4°C and the supernatant obtained was used as enzyme source. The enzyme solution was pre-incubated with various concentrations of compound 7 (5–25 µM) or acarbose (5 µM) or combined [5 µM acarbose with increasing concentrations of compound 7 (1–10 µM)] in 100 mM phosphate buffer pH 6.9 at 37°C for 10 min and the reaction was started by adding maltose (37 mM) or sucrose (56 mM) and incubated at 37°C for 30 min (for maltase) and 60 min (for sucrase). After the respective incubation period, reaction was terminated by keeping the samples in boiling water bath for 10 min. The concentration of glucose released from the reaction mixtures was determined by using Glucose oxidase (GOD POD) kit according to the manufacturer’s protocol. Results were expressed as percentage inhibition of intestinal maltase/sucrase activity.

### Oral maltose and sucrose tolerance test

The experimental animals were randomly divided into 11 groups each consisting of 5 rats. Following overnight fasting, animals were assigned to the following groups and treated with the respective compounds through oral gavage: Group - I Saline control (0.9% saline); Group - II Maltose control (3 g/kg); Group - III Sucrose control (3 g/kg); Group -IV Acarbose (3 mg/kg) + Maltose; Group - V Acarbose (3 mg/kg) + Sucrose; Group - VI Compound 7 (50 mg/kg) + Maltose; Group - VII Compound 7 (100 mg/kg) + Maltose; Group - VIII Compound 7 (50 mg/kg) + Sucrose; Group - IX Compound 7 (100 mg/kg) + Sucrose; Group - X Acarbose (3 mg/kg) + Compound 7 (50 mg/kg) + Maltose; Group –XI Acarbose (3 mg/kg) + Compound 7 (50 mg/kg) + Sucrose. 5 minutes following compound 7 or Acarbose administration either maltose (3 g/kg) or sucrose (3 g/kg) solution were administered to the respective groups. Blood was collected from the tail vein to the tubes containing anticoagulant (2.5% trisodium citrate and 1.37% citric acid in the ratio 1∶5; anti-coagulant: blood) at time point 0 (just before sucrose/maltose administration), and subsequently at 30, 60, 90, 120 and 180 min after substrate (sucrose/maltose) administration. Plasma was separated by centrifuging the samples at 2000 rpm for 10 min and stored at −20°C until analysis. Plasma glucose concentration was determined by using the Glucose oxidase (GOD POD) kit according to the manufacturer’s protocol.

### Glucose uptake study

Porcine diaphragm was purchased from a slaughter house, and cleaned using ice cold 0.9% saline several times to remove blood stains. This diaphragm was used for glucose uptake and inhibition by compound 7. Diaphragm (100 mg) was suspended in a 24 well culture plate containing 500 µL saline. In order to initiate the reaction, 5 mM glucose was added to each well. To enhance the glucose uptake by the diaphragm, 1 unit of insulin was used in each well, and the volume was made up to 1 ml with saline. For inhibition studies, compound 7 at a concentration of 50 & 100 µM was used. From each well 100 µL of the assay mixture was aspirated at different time intervals (0, 5, 10, 20, 30 and 60 min). From this, glucose concentration was measured using Glucose oxidase (GOD POD) kit according to the manufacturer’s protocol.

### Molecular docking studies

The software Insight II/Discovery Studio 2.5 from Accelrys was used for docking and visualization of the results as described earlier [16]. The crystal structure of amylase was retrieved (PDBID: 3TOP). Before performing the Ligand fit protocol of Discovery Studio, the protein was cleaned, and the size and spatial orientation of the active site was identified. All energy calculations were performed using the CHARMM force field. Each energy-minimized final docking position of the ligands was evaluated using the interaction score function in the Ligand Fit module of Discovery Studio as reported previously [17].

### Chem-informatics analysis

Utilizing the available amount of bioactivity data, we also rationalized the modes-of-action for the experimentally tested benzoxazines using *in silico* target prediction approaches. To this end, the Parzen-Rosenblatt Window classifier was employed with the smoothing parameter set to 0.9, using approximately 190,000 bioactive compounds covering 477 human protein targets as the training dataset. For details on the method, dataset and validation see reference 15.

### Statistical analysis

Results are expressed as mean values ± SEM of three independent experiments. Data were compared by analysis of variance (ANOVA) followed by the Tukey “honestly significantly different” (HSD) *post hoc* analysis. Significance was accepted at *p*<0.05 (*), *p*<0.01 (**) and *p*<0.001 (***).

## Results and Discussion

This work is based upon the previous synthesis of an oxazine derivative which was able to mimic the pyranoside structure of glycans functionally [12]. In continuation of the glycobiological aspects, the one-pot syntheses of novel 1,3-benzoxazine scaffold was carried out using 2-aminobenzyl alcohols and different aldehydes in chloroacetic acid *via* aerobic oxidative synthesis (**Fig. 2**). The above design principles led to the synthesis of 13 compounds (**Table 1**) whose α-glucosidase inhibitory activity was validated both *in vivo* as well as *in vitro*, and supported by computational approaches as described below. The synthesized aglycones inhibited both α-glucosidase and α-amylase activity, with overall relatively similar IC_50_ values between 11 µM and 60 µM (**Table 1**). Among the tested derivatives, compound 7{(4-(7-chloro-2,4-dihydro-1H-benzo[d][1,3]oxazin-2-yl)phenol} exhibiting strong inhibition of both glucosidases, with an IC_50_ values of 11 µM and 11.5 µM for α-amylase and α-glucosidase respectively. The addition of phenolic and halogen substituents to the 1,3-benzoxazine ring was found to increase the inhibitory potency (compound 7), whilst the incorporation of a flavone moiety decreases the inhibitory potency (compounds 5, 10, and 13). 1,3-benzoxazines bearing an electron withdrawing chlorine substituent were found to be more potent against α-glucosidase (compound 7), whereas the electron donating methyl group was not particularly favoured (compound 11). Introduction of an imidazole ring (compound 6), to give 1,3-benzoxazine, resulted in an enhanced inhibition (IC_50_ = 16 µM), while a chromene moiety decreased the activity (compound 13).

**Table 1:** Physical characteristics and inhibitory activities (α-glucosidase and α-amylase) of novel 1,3-benzoxazines.

Sl No.	Benzoxazines	Yield	Melting Point	α-Glucosidase IC_50_ (µM)	α-Amylase IC_50_ (µM)
1	4-(2,4-dihydro-1H-benzo[d][1,3]oxazin-2-yl)phenol	83%	160-162°C	17.1±0.1	20.4±0.2
2	2-(1H-indol-3-yl)-2,4-dihydro-1H-benzo[d][1], [3]oxazine	82%	65-67°C	32.0±0.2	59.2±1.0
3	2-(2-methyl-1H-indol-3-yl)-2,4-dihydro-1H-benzo[d][1], [3]oxazine	87%	70-72°C	21.6±0.1	10.6±0.3
4	2-(1-(4-(2-cyanophenyl)benzyl-1H-indol-3-yl),2,4-dihydro-1H-benzo(d)(1,3) oxazine	86%	122-124°C	NS	NS
5	3-(2,4-dihydro-1H-benzo[d][1], [3]oxazin-2-yl)-4H-chromen-4-one	89%	142-144°C	NS	23.3±0.5
6	2-(2-butyl-4-chloro-1H-imidazol-5-yl)-2,4-dihydro-1H-benzo[d][1], [3]oxazine	86%	80-82°C	16.7±1.0	18.5±0.1
7	4-(7-chloro-2,4-dihydro-1H-benzo[d][1], [3]oxazin-2-yl)phenol	87%	139-141°C	11.5±0.1	11.0±0.3
8	7-chloro-2-(1H-indol-3-yl)-2,4-dihydro-1H-benzo[d][1], [3]oxazine	85%	125-127°C	27.7±1.0	26.4±0.5
9	6-chloro-2-(2-phenyl-1H-indol-3-yl)-2,4-dihydro-1H-benzo[d][1], [3]oxazine	89%	220-222°C	27.8±0.2	22.2±0.1
10	3-(6-chloro-2,4-dihydro-1H-benzo[d][1], [3]oxazin-2-yl)-4H-chromen-4-one	83%	141-143°C	31.9±0.8	NS
11	6-methyl-2-(2-methyl-1H-indol-3-yl)-2,4-dihydro-1H-benzo[d][1], [3]oxazine	80 %	102-104°C	23.8±0.1	NS
12	6-methyl-2-(2-phenyl-1H-indol-3-yl)-2,4-dihydro-1H-benzo[d][1], [3]oxazine	86%	220-222°C	20.5±0.5	17.6±0.3
13	3-(6-methyl-2,4-dihydro-1H-benzo[d][1], [3]oxazin-2-yl)-4H-chromen-4-one	88%	202-204°C	NS	51.0±0.3
Acarbose				4.3±0.04	4.4±0.02

NS-not significant.

**Figure 3:**
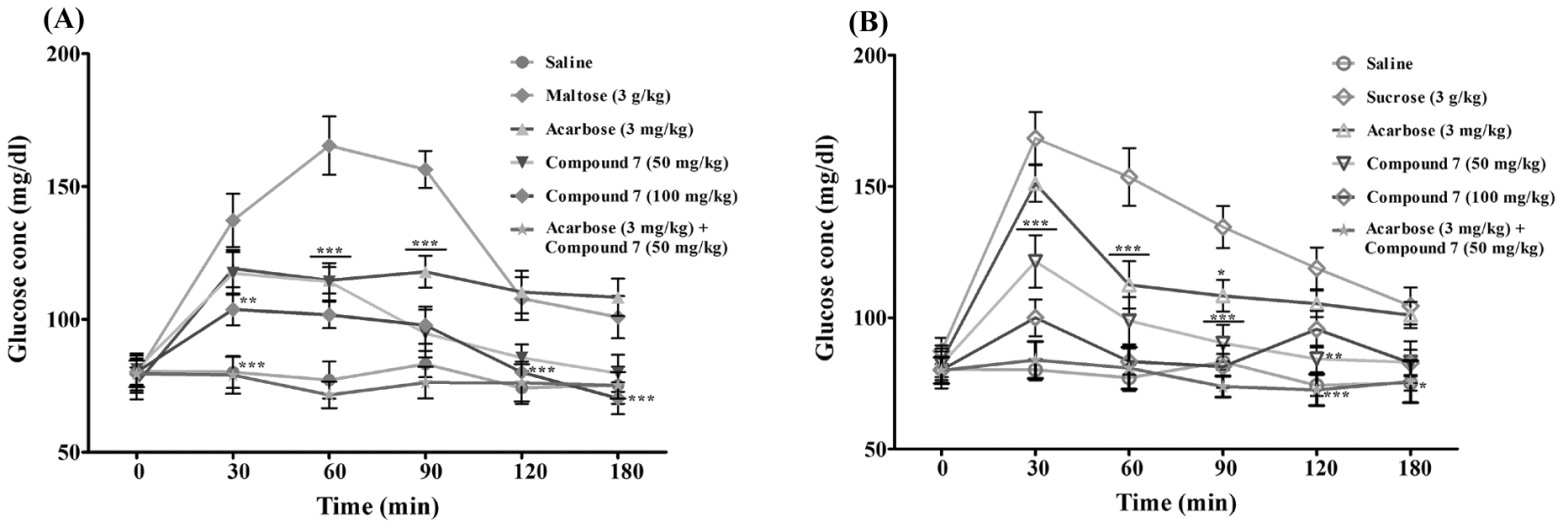
*In vivo* effect of compound 7 {4-(7-chloro-2,4-dihydro-1H-benzo[d][1,3]oxazin-2-yl)phenol} and acarbose on plasma glucose concentration by oral (A) maltose and (B) sucrose tolerance test. Values are presented as mean ± SEM (n = 5). *p<0.05, **p<0.01, ***p<0.001 significant compared to respective maltose/sucrose alone treated groups.

**Figure 4:**
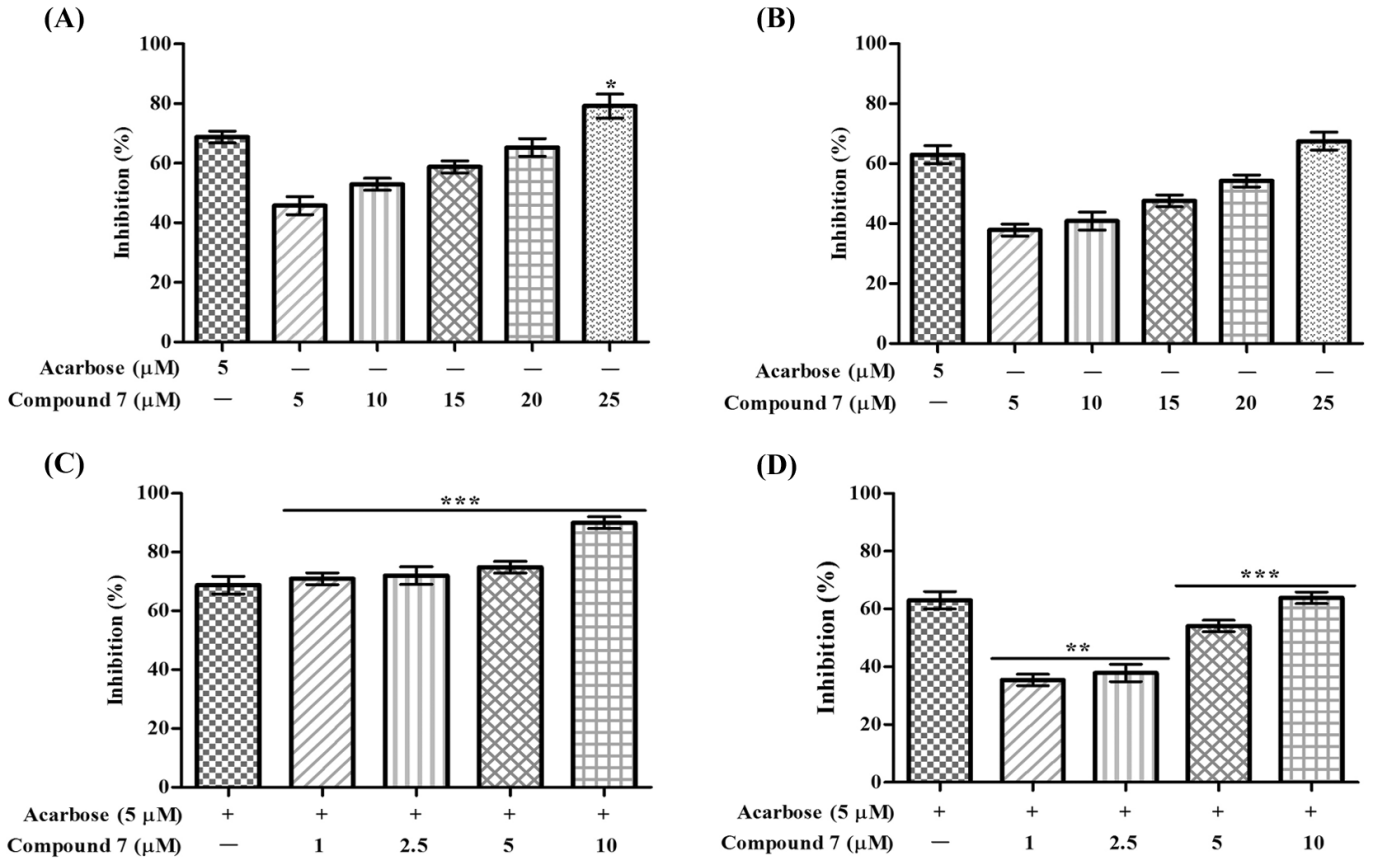
*In vitro* effect of compound 7 and its synergistic effect with acarbose on rat intestinal maltase and sucrase activities. Percentage inhibition of compound 7 on intestinal (A) maltase and (B) sucrose. Percentage inhibition of intestinal maltase (C) and sucrase (D) induced by the compound 7 in presence of acarbose. Percentage inhibition is presented as mean ± SEM of three independent experiments. *p<0.05, **p<0.01, ***p<0.001 significant compared to acarbose.

To study the efficacy of the potent α-glucosidase inhibitor, compound 7 was tested for *in vivo* maltose and sucrose tolerance test on overnight fasted experimental rats by taking acarbose as positive control as well as glucose uptake by porcine diaphragm by using insulin as enhancer. It can be seen that acarbose (3mg/kg) significantly reduced the plasma glucose concentration at 30, 60, 90 min time intervals in starved rats treated with maltose as substrate compared to maltose control (**Fig. 3A**). In sucrose fed rats differences are less pronounced and were only significant at 60 and 90 min time points (**Fig. 3B**). At a concentration of 50 mg/kg body weight, compound 7 inhibited glucose uptake in rats fed with maltose, which was similar to acarbose treatment at 30 and 60 min. However, compound 7 significantly reduced plasma glucose concentration at the 90 min time point compared to acarbose, indicating a different Pharmacokinetic/Pharmacodynamic (PK/PD) profile of compound 7 on between these substrates. Furthermore, compound 7 significantly reduced the plasma glucose concentration throughout all time points (0–180 min) compared to the acarbose treated group when sucrose was used as a substrate (**Fig. 3B**). At a concentration of 100 mg/kg bodyweight, compound 7 was significantly more effective than the acarbose standard at all time points. In order to establish possible synergistic effects between compound 7 and acarbose, plasma glucose levels were measured in starved rats fed individually with maltose and sucrose at different time points up to 180 minutes. In both cases, synergistic activity of compound 7 (50 mg/kg bodyweight) and acarbose (3 mg/kg bodyweight) prevented substrate utilization, and the plasma glucose concentration remained unchanged when compared to the saline treated group. The inhibitory efficacy of compound 7 on rat intestinal glucosidases (maltase and sucrase) was also evaluated. The compound showed both maltase and sucrase inhibitory activities in a dose-dependent manner (**Fig. 4A and 4B**). Acarbose inhibited both the intestinal glucosidases activity with an IC_50_ value of 3.5 µM (maltase) and 4 µM (sucrase), while compound 7 was found to be two-fold selective for maltase (IC_50_ = 10 µM) over sucrase (IC_50_ = 20 µM). Furthermore, acarbose and compound 7 synergistically inhibited intestinal maltase more efficiently compared to intestinal sucrase (**Fig. 4C and 4D**).

**Figure 5:**
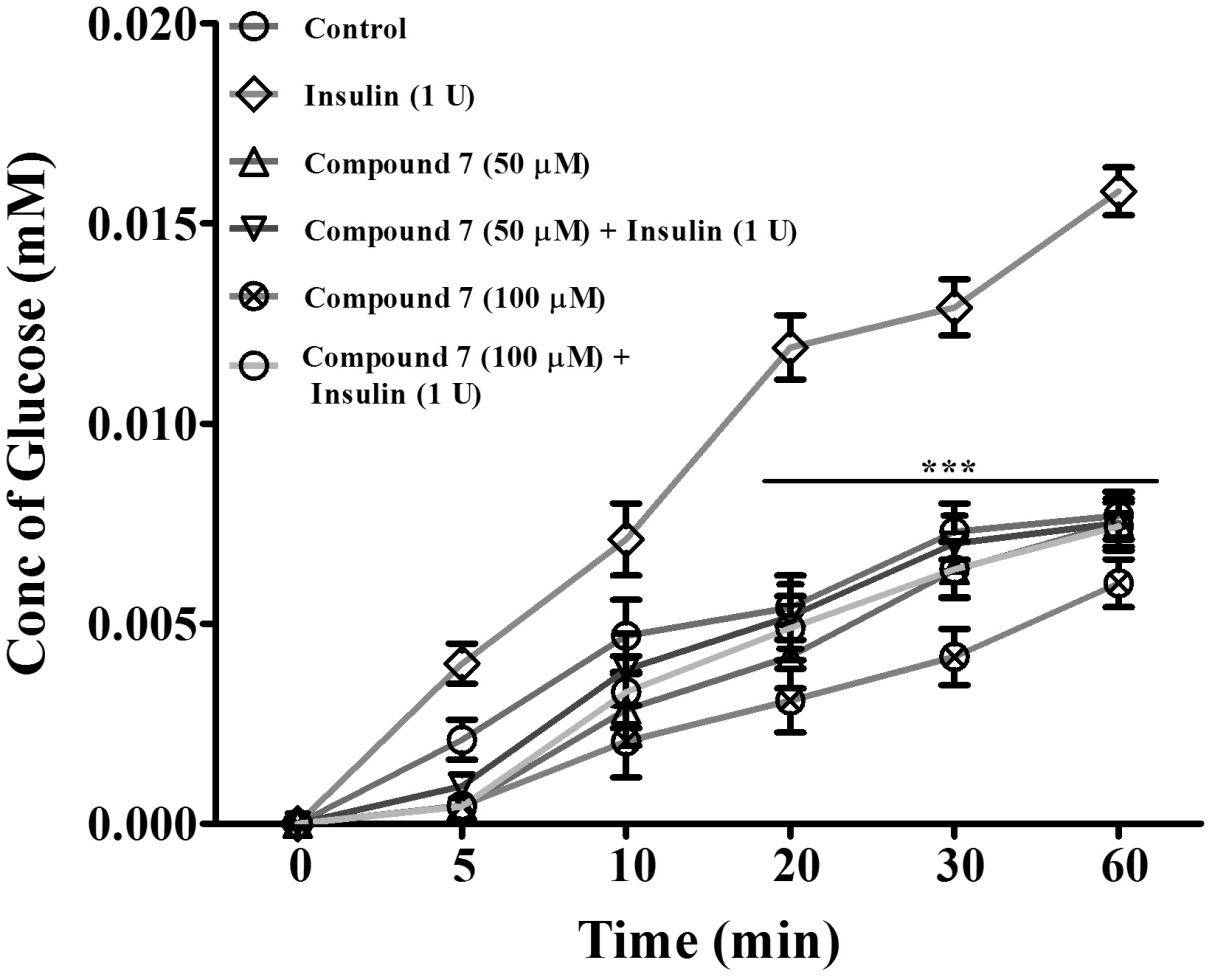
Effect of compound 7 on glucose transport across porcine diaphragm in the presence and absence of insulin. Values are presented as presented as mean ± SEM of three independent experiments. ***p<0.001 significant compared to insulin alone treated diapharagm.

Further, porcine diaphragm was used in order to understand the effect of compound 7 on insulin mediated glucose uptake *via* GLUT4. The glucose transporter isoforms GLUT4 and GLUT1 are highly expressed in muscle cells, with GLUT4 being more abundant in an intracellular compartment from which it is quickly translocated to the plasma membrane as a response to insulin challenge. Both insulin-dependent and non-insulin-dependent diabetes were shown to reduce glucose utilization in muscle either due to a defective expression or dysregulation in GLUT4 translocation [3], [18]. In the present study, glucose uptake was found to be normal in the control group treated with no insulin. However as expected, significant increased glucose uptake was observed in diaphragm treated with insulin (1 U) compared to control. We found that compound 7 at a dose of 50 and 100 µM significantly blocked the glucose uptake both in control and insulin treated diaphragm in a dose-dependent manner (**Fig. 5**).

In addition to *in vivo* and *in vitro* experimental validation of the intended target of α-glucosidase shown above, *in silico* target prediction [19], [20] was performed with the full set of 13 synthesized compounds, in order to obtain a more comprehensive impression of the bioactivity profile of the synthesized 1,3-benzoxazine derivatives. It was found that only compound 7, the most active in the series, is predicted to target the sodium/glucose co transporters 1 and 2, which may contribute to the *in vivo* efficacy of this compound. However, no experimental validation of this additional target has been performed.

**Figure 6:**
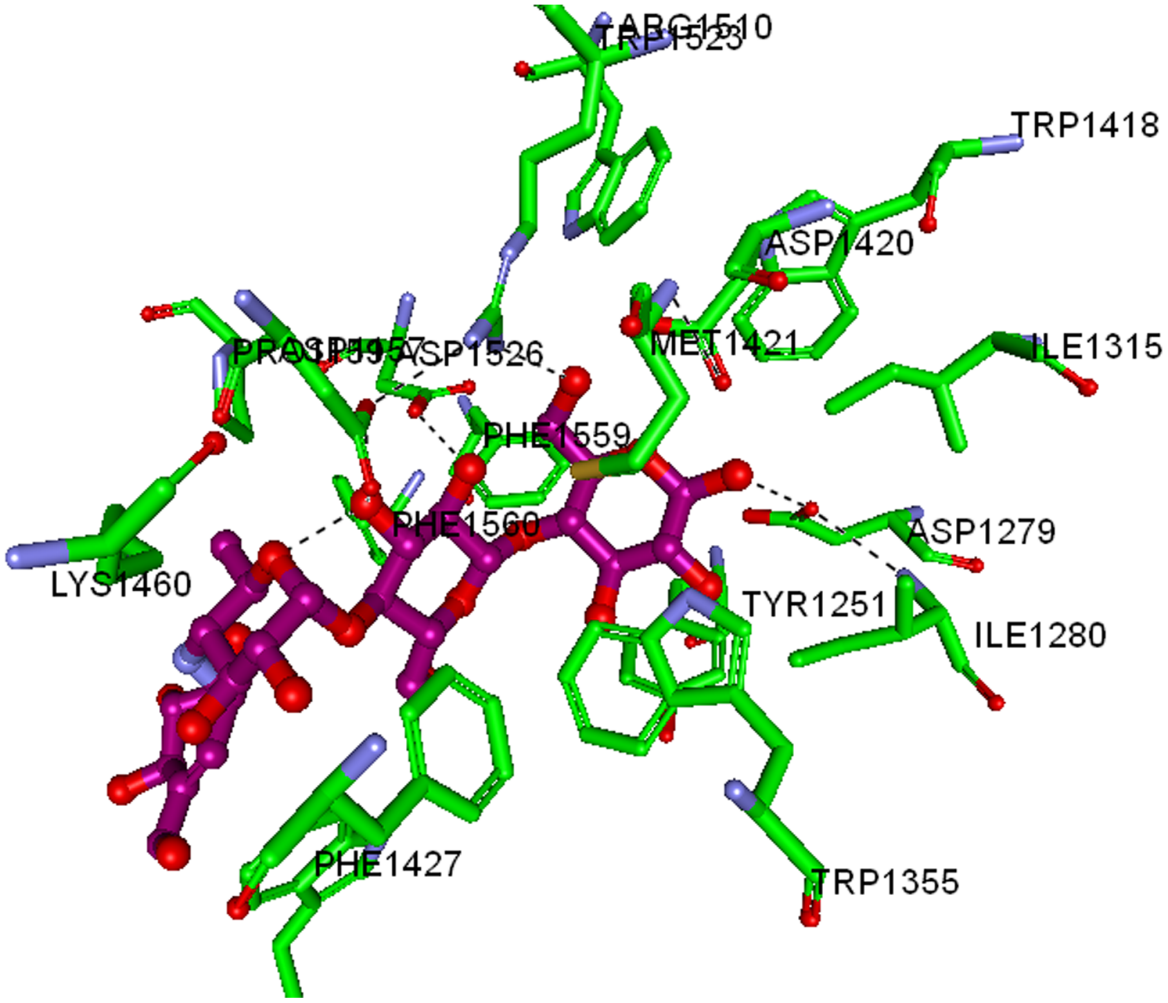
Interaction map of MGAM-C domain co-crystallized with acarbose. The labelled key amino acids are represented as a stick model with the carbon atom as green, and other atoms in their parent colours. The binding of acarbose, whose carbon atom is coloured in pink and other atoms with their parent colour. The hydrogen bonding is represented as dark dotted line.

In order to hypothesize a binding mode, molecular docking has been performed between compound 7 and maltase-glucoamylase (**Fig. 6**).The crystal structure of MGAM-C (Human maltase-glucoamylase C terminal domain) in complex with its inhibitor Acarbose (PDB ID: 3TOP) was used as a model to determine the molecular interaction between enzyme and the synthesized 1,3-benzoxazine derivatives [21]. In MGAM, both the N- and C-terminal domains (MGAM-N and MGAM-C) carry out the same catalytic function with different substrate specificities. The MGAM-C hydrolyzes linear α-1,4-linked oligosaccharide substrates and plays a pivotal role in the production of glucose in the human lumen and considered as an efficient drug target for T2-DM. Since, there is no information regarding the co-crystal structure of murine glucosidase and acarbose, we have used the co-crystal structure of MGAM-C and acarbose for docking studies.

**Figure 7:**
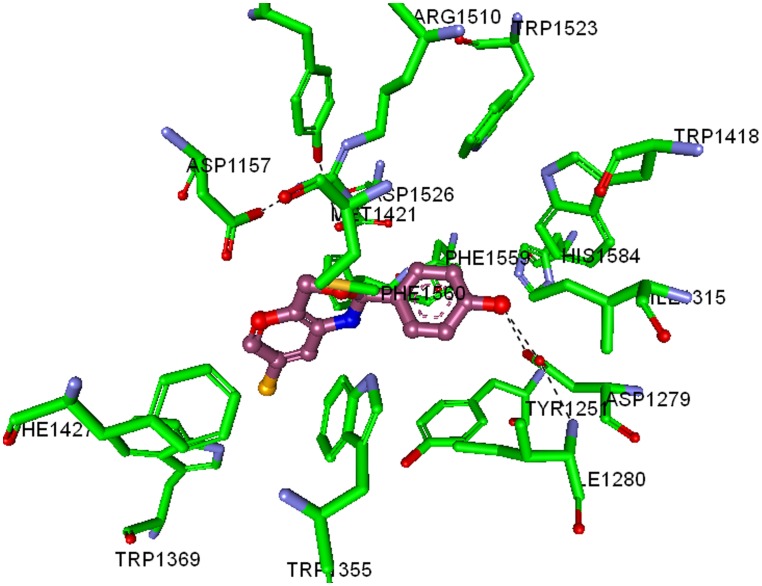
Interaction map of MGAM-C catalytic domain co-crystallized with Compound 7. The labelled key amino acids are represented as a stick model with carbon atom as green, and other atoms with their parent colour. The binding of compound 7, whose carbon atom is coloured in pink and other atoms with their parent colour. The hydrogen bonding is represented in dark dotted line.

The synthesized 1,3-benzoxazine derivatives have relatively similar IC_50_ values, Dock Score (column ‘DS’) in particular seems to show higher scores for the more active compounds (7, 1 and 6) as opposed to less active compounds (2, 8, 9 and 10) (**Table 2**). Structurally, the most active compound 7, binds deep in the MGAM catalytic domain (**Fig. 7**), in which the chloro- benzoxazine ring stacks into the hydrophobic cluster of Tyr1251, Trp1355, Trp1369, Tyr1427, Phe1559, and Phe1560, along with the phenolic ring stacked to Tyr1251, His1584, Trp1418, and Trp1523. The terminal hydroxyl group of the phenolic component of compound 7 shows hydrogen bonding with Asp1279 and Ile1280, which are also involved in hydrogen bonding with the terminal hydroxyl group of Acarbose in the co-crystal. In addition, the exposed oxygen atom in the benzoxazine ring of compound 7 appears to show ionic interaction with Asp1526 and Arg1510, which is also crucial in the Acarbose-MGAM co-crystal. These results clearly suggest that both acarbose and compound 7 shares similar binding pattern towards MGAM-C.

**Table 2:** Molecular docking results of MGAM-C with 1,3-benzoxazine derivatives.

Compounds	Mol Wt	LS1D	LS2D	PLP1	PLP2	JAIN	-PMF	-LE	DS
1	227.2	2.42	3.94	56.46	52.2	1	166.72	2.1	56.0
2	250.2	3.03	4.78	58.76	59.46	2.47	181.93	3.0	50.2
3	264.3	1.6	4.6	70.0	66.6	2.3	176.1	2.9	51.6
4	441.5	2.82	5.41	79.78	76.05	2.79	162.71	6.6	64.9
5	279.2	2.23	4.85	71.4	66.83	1.99	187.08	3.0	50.6
6	291.7	3.31	5.4	74.34	73.9	2.6	156.19	3.0	56.8
7	261.7	2.94	4.7	64.7	59.09	1.25	168.66	−2.5	58.9
8	284.7	1.58	4.31	64.8	63.17	1.71	168.13	3.4	52.7
9	360.8	1.49	4.76	73.11	71.34	2.03	188.54	6.6	58.2
10	313.7	3.11	5.13	75.77	72.83	2.12	191.14	2.9	52.3
11	278.3	1.48	4.38	63.1	62.07	2.33	164.55	3.8	51.6
12	340.4	1.72	4.62	68.11	69.58	3.37	181.81	6.3	57.4
13	293.3	2.23	4.58	72.74	68.95	1.34	198.94	1.6	51.9
Acarbose	602.2	5.88	5.84	63.24	60.17	−1.73	270.87	−9.4	64.3

LS1D and LS2D: LigScore1D and 2D are a fast, simple, scoring function for predicting receptor-ligand binding affinities.

PLP1 and PLP2: Piecewise Linear Potentials 1 and 2 are fast, simple, docking function that has been shown to correlate well with protein-ligand binding affinities.

JAIN: An empirical scoring function (lipophilic interactions, polar attractive interactions, polar repulsive interactions, solvation of the protein and ligand, and an entropy term for the ligand) through an evaluation of the structures and binding affinities of a series of protein-ligand complexes.

PMF: Potential of Mean Force is the scoring function developed based on statistical analysis of the 3D structures of protein-ligand complexes.

LE: Ligand internal Energy, the internal non bonded ligand energy is calculated for each new conformation that is generated.

DS: Dock Score, candidate ligand poses are evaluated and prioritized according to the Dock Score function.

Finally, we applied a metabolite prediction software, namely MetaPrint2D-React [22], to bioactive compound 7 and found the most likely metabolic site to be a glucuronidation site with a (significant) normalised occurrence ratio of less than 0.33 and but greater than 0.15. Hence, this study for the first time demonstrated the design, synthesis, and characterization of novel 1,3-benzoxazine aglycones and their validation *in vitro* and *in vivo*. Compound 7 significantly inhibited rat intestinal glucosidases, namely maltase and sucrase, in a dose-dependent fashion and led to decreased blood sugar levels in starved rat model. In addition to this, compound 7 acts synergistically with Acarbose in lowering the blood sugar levels to that of the saline control alone.

## Summary

This study demonstrated the novel synthesis of benzoxazine glycones and their effective inhibition towards glucosidases. The newly synthesized 1,3-benzoxazine derivatives showed better IC_50_ values for both α-glucosidase and α-amylase, ranging from 11–60 µM, and are found to be effective when compared to natural substrate aglycones, such as BOA and derivatives that deteriorate fatter in aqueous solution. The *in silico* molecular docking studies revealed that benzoxazines bind to the catalytic domain of MGAM-C, correlating with a high DS for the most active compound 7. The docking score of compound 7 and binding poses were found to be similar with the anti-diabetic drug acarbose. Furthermore, studies of *in silico* target prediction algorithms showed that compound 7 potentially targets the sodium-glucose cotransporter 1. Both *in vitro* and *in vivo* experimental results suggested an anti-hyperglycemic effect of compound 7, which significantly inhibits glucose uptake in starved rat model by blocking intestinal maltase and sucrase. Evidently, compound 7 was determined to possess the glucuronidation site, which potentially converts it into a stable glycoside *in vivo*. The aglycones synthesized in this study could hence constitute a novel pharmacological starting point for the treatment or alleviation of T2-DM and its secondary complications. However, further studies elucidating interaction between compound 7 and specific glucose transporters would be highly exciting.
